# Early- and mid-career transition to research leadership in Africa

**DOI:** 10.12688/wellcomeopenres.16540.1

**Published:** 2021-04-07

**Authors:** Linda Mtwisha, Jose Jackson, Alison Mitchel, Ama de-Graft Aikins, Harriet Kebirungi, Karim Outtara, Clare Viney

**Affiliations:** 1 Research Division, University of Cape Town, Cape Town, 7700, South Africa; 2 Alliance for African Partnership, Michigan State University, East Lansing, Michigan, 48824, USA; 3 Department of Diversity and Inclusion,, Lincoln University, Lincoln, LN6 7TS, UK; 4 Institute of Advanced Studies, University College London, London, WC1E 6BT, UK; 5 Gender Mainstreaming Unit, Kyambogo University, Kampala, Uganda; 6 Swiss Center for Scientific Research, Abidjan University, Abidjan, Cote d'Ivoire; 7 Careers Research and Advisory Centre (CRAC) / Vitae, Cambridge, CB5 8LA, UK

**Keywords:** Transition, early career researchers, mid-career researchers, research leadership, professional development, Africa

## Abstract

This article examines the early-and mid-career transition to research leadership in Africa. Much of the available African literature on research leadership indicate several challenges related to poor conceptualisations of career transitions and gaps in the availability of research training. Qualitative data were collected using individual interviews (n=24) and focus groups (n=27) to identify key transition points of early career researchers (ECRs) and mid-career researchers (MCRs) in selected African countries. The qualitative data was complemented with quantitative survey questionnaires (n=250) and a triangulation approach was adopted to analyse the results. The findings were themed into different categories describing the common career paths, stages and challenges of research leaders. The latter part of the findings present a discussion on development approaches to attract and retain researchers in African universities. By focusing on the African continent, this study contributes to the current body of literature on research leadership in the Global South.

## Introduction

Although studies on early career researchers (ECRs) and mid-career researchers (MCRs) have received significant attention in the Global North ( Baldwin *et al.,* 2008; Clay, 2012; European Commission, 2018; Hottenrott & Lawson, 2017; Hurley & Taylor, 2016; Wong, 2019), the terms remain poorly understood in higher education institutions (HEIs). Australian research typically associate the early career period with research capability in the first five years following the completion of a doctoral programme ( Bosanquet *et al.,* 2017; Browning *et al.*, 2017; Hurley & Taylor, 2016; Schriever & Grainger, 2019) while the African literature classify ECRs as assistant lecturers, lecturers or occasionally senior lecturers ( Harle, 2011; Merritt *et al.*, 2019; Shinkafi, 2020). The European Commission funded project called the MORE _3_ study provided insight into career progression for researchers and also the four career stages at HEIs in Europe ( European Commission, 2018). Given that no universally accepted definition for ECRs exist, the mid-career period (transition stage following ECR) is also controversially conceptualised.

In the same vein, Owusu *et al.* (2014:1) maintains that there is a general lack of agreement on the definition of research leadership and a ‘particular need for a definition in the African context’ is essential. Evans (2014:46) regards research leadership as ‘the influence of one or more people on research-related behaviour, attitudes or intellectual capacity of others’. Evans further distinguishes three specific features that characterise research leadership: influence that enhances people’s capacity to make appropriate choices, to achieve requisite standards, and to effect processes, within research activity ( Evans, 2014: 46). Several other scholars consider research leadership as leading with new knowledge, while others suggest that it depends on the number of staff, number of publications, global recognition or running a research centre ( Fraser *et al.,* 2017; Owusu *et al.*, 2014; Richards *et al*., 2021).

In this article, research leaders are defined as individuals who are established in their research field, leading and running large research groups and large research facilities ( Vitae, 2020). There is a consensus that research leaders are at the forefront of their field in terms of publication quality and numbers, attract large research grants, supervise and mentor graduate students and successfully implement large-scale research programmes ( Vitae, 2020). The article further recognises that research leadership involves a continuous learning journey with pre-defined stages and transition points: early career (doctoral candidates, doctoral graduates and postdoctoral fellows) and mid-career (managing small research teams). In Africa, little evidence exists on the styles and perspectives of leadership within research contexts. The outcomes confirm that African research leadership can be considered a combination of leading self, leading others and leading research excellence ( Vitae, 2020: 11).

Since researchers are a relatively scarce resource in the African context ( Vitae, 2020:8), this study focuses on the transition pathways of ECRs and MCRs to research leadership using a mixed-method approach. In doing so, the aim of the article is to examine the common career paths, stages and challenges faced by research leaders in several African countries and ultimately propose development approaches to attract and retain researchers in academia. The study forms part of a larger project undertaken by the Wellcome and Alliance for Accelerating Excellence in Science in Africa (AESA), which aimed to support the capacity-building capabilities for researchers within the African continent. Research leaders play an essential role in influencing, transforming and strengthening institutional, national and international research systems. The African continent is of particular interest, as little evidence exists on the styles and perspectives of leadership within research contexts. Despite the wealth of research on leadership in the Global North, the themes cannot be extrapolated to the African context, as it is considerably diverse in cultural beliefs, practices, infrastructure, systems and resources.

The article is structured as follows: the first section of this article provides a brief literature review on research leadership globally and common transition challenges of ECRs and MCRs. Next, the methodology for the mixed-method approach adopted in this study is provided. The Results and discussion section details the themes that emerged and positions the contribution of this study within existing scholarship.

## Literature review

Though a breadth of knowledge on career transitions exists ( Bosanquet *et al.*, 2017; Frick *et al.*, 2017; Merritt *et al.*, 2019; Schriever & Grainger, 2019; Sullivan & Ariss, 2021), literature on the pathways of early and mid-career researchers (EMCRs) remain fragmented. Research suggests that typical challenges facing ECRs relate to scarce grant opportunities for national and international conferences ( Fraser *et al*., 2017; Harle, 2011), difficulty locating experienced supervisors and mentors ( Richards *et al.*, 2021; Schriever & Grainger, 2019), high dropout rates among doctoral candidates, and lengthy completion of studies ( Browning *et al*., 2017). In addition, understanding the destinations and career paths of doctoral graduates and how they contribute to society, culture and economy is also important ( Vitae, 2020). Moreover, Hurley & Taylor (2016) draw attention to a potential synergy between two issues: the oversupply of doctoral graduates exacerbating the challenges facing ECRs and the imbalance between research and practice. Their research suggest that doctoral graduates discontinue their careers in academia due to the lack of opportunities despite the culture of practice ( Hurley & Taylor, 2016).

As a starting point to understand the early career transition of researchers, Williams *et al.* (2017:73) propose a concept of ‘making it’ as it captures two key ideas. The authors refer to the first idea as how to thrive, or ‘make it big,’ in a modern university, while the other is about how to survive, or ‘make it through,’ the trials and tribulations of academic life ( Williams *et al.*, 2017). A common indicator of success in the extant literature is the culture of ‘publish or perish’ ( Hurley & Taylor, 2016). In order to attain leadership positions, grants or promotions, the general directive is ‘publish or perish’, which is a critical measure of research success, particularly if submitted to quality academic journals ( Hurley & Taylor, 2016:9). According to Browning *et al*. (2017:361), the success of ECRs remains highly dependent on the notion of ‘survival of the fittest’ in most HEIs.

Research on MCRs indicate that the success and transition to research leadership is considerably diverse from ECRs and pose distinctive challenges that colour the nature of academic life during this period ( Fraser *et al.*, 2017; Wong, 2019). For MCRs, some of the transitional challenges include issues of increased workload, career development, less faculty attention, feelings of neglect, the pressure to remain competitive, short-term contracts and dealing with the rejection of manuscripts ( Acola, 2012; Baldwin *et al*., 2008; Browning *et al.*, 2017; Wong, 2019). Research by Harle (2011) suggests that the gap between doctorate degrees and the mid-career needs to be bridged more effectively to ensure that the (a) research momentum is not lost post-doctorate period; (b) research is capitalised on to provide a firm foundation for future work, and (c) to harness the value and potential which existing relationships can offer.

Literature on research leadership in public Institutions of Higher Learning ( Acola, 2012; Browning *et al*., 2017; Clay, 2012), indicates that there remains little to no attention given in providing formal research leadership training in a number of these institutions. An Australian study that focused on women in academia ( Dever *et al.*, 2006) found that factors such as passion, reputable and established networks, mentorship, participating in consortium research, undertaking supervisory responsibilities and most importantly maintaining limited administrative responsibilities are key to research leaders’ success. In this study, however, men have greater opportunity to gain the ‘right’ experiences, partly as a result of the male research tradition. In this study, it was clear that women were not given the same opportunities to gain leadership experience as men.

Statistical data from UNESCO (2017) suggests that only a quarter of academic staff in tertiary education across Sub-Saharan Africa (SSA) are women. There is a noticeable variation between countries in SSA from 37% in Botswana to less than 10% in many ( UNESCO, 2017). Gender equity is often hampered by cultural expectations and lack of governmental and institutional support. The data further suggests that the global average of researchers per million inhabitants is 1478. For example in Africa, only Tunisia (2000 researchers) exceeds the average. Below the average, Morocco is closest (1100 researchers) followed by Egypt (680 researchers), Senegal (550 researchers) and South Africa (494 researchers). In multiple countries in SSA, the average is fewer than 50 researchers per one million inhabitants.

Most HEIs prioritise teaching and the expansion of universities has generally focused on undergraduate and master’s levels rather than doctorates ( UNESCO, 2017). Despite these resource challenges, SSA’s share of the global output in research papers increased from 0.44% in 2003 to 0.72% in 2012. Intra-regional collaboration between researchers of African countries accounted for below 15%, thus indicating that networked communities of academics are still exceptional. An increase in doctorate enrolments seen in some countries is driven by government policies to raise qualification levels in HEIs. Such policies are operating at different speeds. For example, in Ethiopia, where only 8% of university staff were doctorate-qualified, doctorate enrolments as a proportion of all HE enrolments has risen to almost 8%. In South Africa, Ghana and Kenya, in contrast, doctorate enrolments form less than 2% of total enrolments. In these countries, doctorate-qualified personnel range from 31% in Ghana and 43% in South Africa. The studies presented thus far, may create the impression that much academic work has been done, but relatively little is known about EMCRs in a low-income country such as Africa.

## Methods

### Study design

Since very little is known about the transitions of EMCRs to research leadership in Africa, a mixed methods approach was adopted to examine the career paths of those who have navigated the transition of ECRs and MCRs. This approach is common in the social sciences to gain a more detailed perspective and to verify the validity of the information provided ( Browning *et al.,* 2017:361). The research paradigm was based on the conception that research leadership involved continuous learning journey on a path with defined stages and transition points: early career (research students and postdoctoral researchers) and mid-career (managing a research team). In-depth interviews and focus groups were conducted and complemented with a quantitative-based survey. The Vitae Researcher Development Framework (RDF), specifically its “leadership lens” was used as the research leadership model in the study. The RDF is based on building competencies through a series of development phases and has been used successfully in a range of international contexts ( Vitae, 2012). A team of scholars in Southern, East and West Africa who had prior experience in qualitative and quantitative research methods and publishing their work collected the data. The results of the interviews, focus groups and survey built on findings from the literature review of research leadership in Africa and a web search of current leadership training and development opportunities for research leaders.

### Participants

The sampling approach involved a range of participants including research leaders, EMCRs, research managers, senior management and funders who were in the health sciences and were largely from institutions in East, West and Southern Africa. A small number of participants were based in North Africa. Most participants were based in Anglophone regions.

In identifying successful research leaders to participate in the interviews, the definition ‘research leaders who are established in their research field, running large research groups, leading large research teams and research facilities’ was used. These included, for example, African Academy of Sciences (AAS) Fellows, National Academy Fellows and DELTAS consortia research leaders. Focus group participants (EMCRs and research managers) were identified through local project team networks in Cote d’Ivoire, Uganda, South Africa and Ghana. Research students as well as researchers, research leaders and research managers were invited to participate in the survey through the networks of the local experts in each participating country, as well as research and innovation management associations across Africa.

### Ethical considerations

Ethical approval to conduct the study was received by the Institutional Review Board, Office of Regulatory Affairs in the Human Research Protection Programme at Michigan State University (STUDY00001318). All participants were provided with information about the study, the research team, including their rights as a research participant and were requested to sign a consent form if they agreed to participate in the study.

### Data collection

The project involved multiple phases and an interviewer guide was developed and used by all researchers. The first phase included a pilot focus group at the DELTAS Annual Conference in Johannesburg in 2018 with eleven senior research leaders, directors and funders to elicit themes and question areas. The data gathering phase involved a literature search to understand the range of perceptions and research about ‘successful research leadership’ in Africa.

In the second phase, 24 semi-structured one-hour interviews with successful research leaders in eight African countries, and two focus groups with a total of 27 participants (Cote d’Ivoire, Ghana, South Africa and Uganda) were conducted by local in-country expert interviewers and facilitators. All interviews were either held in-person or by telephone and were recorded and transcribed by the research lead.

**Figure 1.**
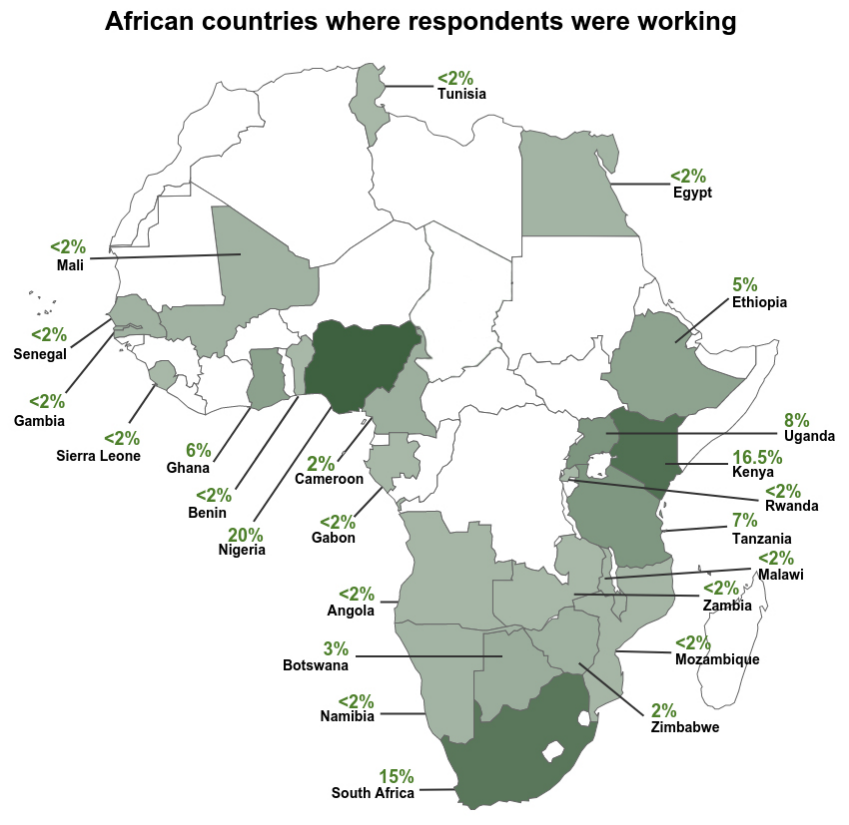
African countries where respondents were working.

The survey questionnaire was distributed to a target response of 250 research students, researchers, research leaders and research managers using Survey Monkey ( Figure 1). The survey questions can be found in the *Underlying data* ( Viney & De-Graft Aikins, 2021a).

### Data analysis

The analysis phase involved a synthesis of findings to develop and recommend potential frameworks for the development of the research leadership in Africa. The creation of case studies of existing leadership provision in Africa that illustrate alignment of provision to researchers’ needs formed part of this phase.

## Results and discussion

The next section describes the themes that emerged from the study primarily based on qualitative interviews and focus groups. The personal motivations, challenges and competencies of participants; personal and institutional actions for gender inclusion; institutional actions needed for transforming the leadership path in African research; and what would have been useful to know along the way to becoming effective leaders of the next generation are outlined next.

### Common career paths of EMCRs to research leadership in Africa

From the interviews, it emerged that the theme of career ownership and planning is an important step towards research leadership in the early stages post-doctorate completion. Participants in this study were intentional about the advancement of their development from the outset and early on in their research careers. This finding corresponds with Browning *et al.* (2017) who contended that the early years post-doctorate are critical for developing a record of accomplishment that can lead to a successful research career. A key realisation of a personal vision and mission for their research led them to strategic choices in career development, often but not necessarily taking them outside Africa at doctoral and (sometimes) postdoctoral stages before returning ‘home’ to be research leaders.

A senior research leader who was intentional about career ownership and planning shared:

*“The decision to come back home was also critical because I could have stayed in the US and still be one of the many research associates or whatever. But, I decided to come back to the University and start my own research group and tried to make a difference.”*

Participants made a distinction between two routes to international recognition: one through becoming a research leader; and the other through becoming an ‘international researcher’. An international researcher was understood as a researcher who has a substantial number of recent high-quality publications in leading international journals and reputable book publishers ( Vitae, 2020). Whether or not they had spent part of their career outside Africa, all interviewees described a strong personal commitment to the African continent.

Consistent with the extant literature ( Bosanquet *et al*., 2017; Browning *et al.,* 2017; Hurley & Taylor, 2016; Moosa, 2020), the narratives of participants in this study reflected the following common transition points and career paths to research leadership in HE: (a) completing a doctorate; (b) gaining postdoctoral experience; (c) getting leadership responsibility and (d) managing a team. In their mid-career stages, researchers led other leaders, and some had moved a stage further to lead organisations such as research institutes. In order to achieve these various career transitions, all participants cited gaining the requisite research capital, with special mention of skills in writing for publication and grant applications and accessing funding. According to Shinkafi’s (2020) recent work on ECRs, funding remains limited in African HEIs and emphasis is placed primarily on senior scholars such as professors and associate professors.

At each career transition stage, the individual needs to change and develop new competencies to undertake new responsibilities. For example, ‘when leading others, the focus of time management shifts from one’s own work to that of others, and alters the relative importance of different aspects of one’s workload’ ( Vitae, 2020:16). This view is particularly applicable to MCRs and corresponds with the research undertaken by Baldwin *et al.* (2008) at Michigan State University. These authors contend that during the mid-career stage, researchers tend to encounter higher expectations that grow substantially in the post-tenure years ( Baldwin *et al.*, 2008). Once they become fully-fledged members, they are expected to assume new roles and duties. Many participants reported they had no training or support in transitions. In areas where they did, commonly mentioned enablers were access to mentors, international opportunities and gaining experience in another sector. Leaders were proactive in making this happen.

Two survey respondents shared the following:

*“Leadership is a process, a learning path, the more exposure one gets …the more chances to keep up and improve.”*

*“It is about a combination of measures not one in isolation measure or short-term initiative.”*

EMCRs shared that navigating cultural and institutional environments influenced all stages of their careers. For the women research leaders in this study, navigating these complex environments also required managing societal expectations and family responsibilities. This finding is consistent with research done by ACOLA, 2012; Baldwin *et al.*, 2008; Fraser *et al.*, 2017. Proactively developing good work relationships with colleagues was important. Curating research profiles and trajectories on academic community platforms such as ResearchGate and Academia.edu was not part of the culture of research capacity building when the senior academics were building their careers and so, when questioned, they felt a disconnect with this culture. Participants concluded that more structured career planning and a proactive approach to building their research profile would have been an added benefit, as would preparation for trying to balance work and personal life.

Research leaders identified key career transition points as they progressed from being an ECR to MCR. It is important that as developing leaders go through each transition stage they are given the opportunity to prepare themselves by reassessing their values, tasks and time, and to transition from personal focus to community focus and the ‘common good’ (teamwork). Such opportunities were not offered to current research leaders, who had to manage themselves though the process; some reporting that they had never caught up, for example, on work-life balance. Regarding leadership development as a continuous process with transition points where extra time and support are required, would help leaders settle into their roles more effectively.

### Challenges facing EMCRs to research leadership in African HEIs

The importance of individual qualities in research leadership to achieve personal effectiveness were repeatedly highlighted during the interviews. However, achieving personal effectiveness can sometimes be impeded by factors such as institutional barriers and culture, balancing priorities in their academic roles to focus on research, and difficult relationships with collaborators. These factors are also well-documented in the Australian-focused literature ( Bosanquet *et al.*, 2017; Browning *et al*., 2017; Schriever & Grainger, 2019). Other challenges relating to leaders’ African experiences were scholarly isolation, lack of visibility and limited access to ‘like-minded’ researchers. All participants expressed a passion for their research, which helped them through difficult times. As Shinkafi (2020) contend, there is a possibility of ECRs leaving the university at an early stage for a lucrative job outside academia. Elements of personal effectiveness to research leadership development included: continual learning from others; effective multi-directional communication; becoming a role model; and research talent capacity building. As research leaders develop and take on more responsibility, their personal visibility increases and hence their potential as a role model. The mentoring literature on EMCRs suggest that MCRs are more than capable of providing successful and productive mentoring experiences for ECRs ( Schriever & Grainger, 2019).

Another challenge facing EMCRs in African HEIs is self-management. This trait was considered important, especially in work-life balance, time management, and avoiding complacency and becoming out of date. Similar views were expressed in research undertaken by Hottenrott & Lawson (2017) and Williams *et al*. (2017). Solutions offered included taking time for self-reflection, conducting constant personal evaluation with a view to improve, and learning from co-creating projects and wider multi-directional learning. Participants realised that effective learning was also multidirectional. They frequently expressed concerns about being able to find the time for continual self-development, a recurrent theme for participants balancing multiple work roles and personal life. For both ECRs and MCRs in African HEIs, research funding remains a challenge. Funding limitations for ECRs relate to scarce grant opportunities for national and international conferences ( Fraser *et al.,* 2017; Harle, 2011) and securing funding as a MCR is progressively challenging ( Wong, 2019).

A focus group participant shared the following:

*“Poor disciplinary knowledge and inability to guide the student within the disciplinary field…research leaders…constantly pressurized through multiple responsibilities…the pressure associated with the ‘numbers game’ within publishing and student throughput…”*

Key challenges for research leaders were reported as influencing senior management; maintaining reputation and credibility; scarce resources including skilled and knowledgeable colleagues; and lack of financial support to secure international linkages. This clearly differentiate team leadership from full research leadership and can be seen as the additional responsibilities of running research as an ‘organisation’ or business. Managing risk was also highlighted as a key challenge in the group consultations with DELTAS research leaders. Strategies to deal with these challenges variously required awareness, monitoring, problem solving, influencing skills, reflective learning, maintaining personal values, and being creative with limited resources. Interviewees cited challenges of bureaucracy, lack of investment in relevant knowledge and skills, and of mobilising collective effort. Leadership responses required innovative approaches, involving others, and effective communication.

### General characteristics and competencies required for research leadership in Africa

**Figure 2.**
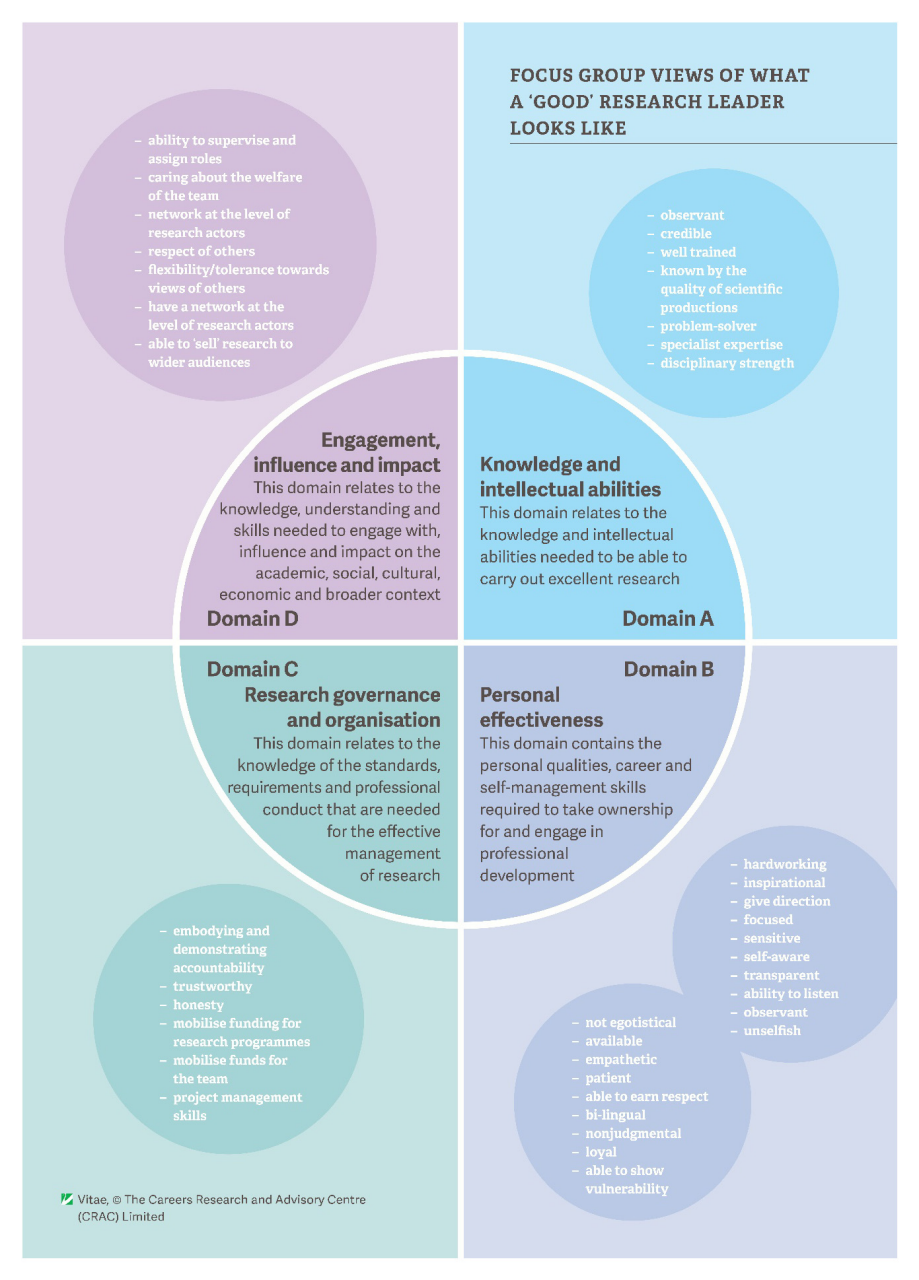
Focus group views on the attributes of a ‘good’ research leader, mapped to the Vitae Researcher Development Framework (RDF) domains.

The overall feedback from participants on the attributes of a good research leader is shown in Figure 2. The significance of impact as a determinant for a research leadership was frequently highlighted during fieldwork in this study. Impact was seen to require global engagement, either as a research leader, or as an internationally recognised researcher, and institutional support necessary in building an international presence. This finding concurred with similar research conducted in Australia (e.g. ACOLA, 2012; Clay, 2012). Participants also underscored the importance of values. These encompassed ‘shared ethics and world views’ between research partners and leading by example within the research team. Focusing on values applied at all levels of engagement, influence and impact – from global to local.

As one participant stated:

*“Providing exemplary leadership by doing to achieve goals that have been set. For example, during bidding, they would come up together with a team to write proposals or manuals with the goal of impacting the region and the community through collaboration with specific goals like manuals on food security and HIV/AIDS. The manuals should be believed by other people rather than imposing ideas on them…”*

Other common qualities required as a research leader included integrity (ethical and principle-led work habits); credibility; vision; relationship-management; developing others; decision fairness; outcome concern; self-awareness; self-management; lifelong learning; mobilising others; results focus. The interview discussions were also centered on the competencies of research leaders. MCRs found the framework useful in thinking about building research capacity and mentoring others towards research leadership. In general, the highest priority competencies were seen to be knowledge base, cognitive abilities, creativity, personal qualities, professional conduct, research management, finance, funding and resources, working with others, engagement and impact. MCRs repeatedly indicated that building a ‘pillar’ of research excellence should be the predominant focus of the start of the path to research leadership. On the later stages, there was general agreement that research leadership is the integration of competencies to deal with complex tasks (subject knowledge). It emerged that EMCRs need to bring together competencies that go beyond the day-to-day research to lead research, researchers, programmes and institutes effectively.

There was consensus that research leadership involved leading a team, leading by example and creating the path for team members to achieve established goals of research; primarily, accessing grants, getting published and getting promoted. ‘Bad’ leadership was often experienced as lack of guidance. This stemmed from the leader’s lack of relevant expertise and/or from inaccessibility due to multiple work pressures. Personal qualities for personal effectiveness are much mentioned and among these, engagement, influence and impact, relational leadership competencies feature strongly. Leadership is relational with no ‘one-size-fits-all’ definition of successful research leadership. Within leadership there is a ‘fit’ to those who led, and research leaders have to look outwards to the wider environment and inwards to lead the people.

### Development approaches for EMCRs to transition successfully into research leadership

Participants in this study shared a number of reflections and advice for the next generation about how to develop research leaders. These development approaches can be categorised as follow: (a) training and development routes, (b) research knowledge and practice, and (c) personal qualities researchers need to develop. Participants recommended that ECRs and MCRs seek out a number of diverse routes to develop their leadership potential. It was repeatedly highlighted that while ECRs build their research profile, they need to be open-minded simultaneously: It is important to give attention to broader aspects of their research (using strategic and critical thinking and ethical sensitivity) and their personal development.

Mentorship and mentorship programmes featured strongly in the discussions of institutional support for research development. This is not an emerging theme and have surfaced in the existing international literature for some time ( Baldwin *et al.,* 2008; Clay, 2012; Fraser *et al.*, 2017; Schriever & Grainger, 2019; Sullivan & Ariss, 2021). International collaboration, formal and structured leadership training, funding for internships, work opportunities or time off from work were mentioned as important measures for future development. It was found that research training schemes for ECRs existed at institute and school level at some HEIs. These schemes implicitly included the goals of training research leaders. Most of these schemes were funded by external funders (usually US or European based), but delivered by local researchers in collaboration, in some cases, with northern partners. The focus was primarily on developing research capability, rather than specifically on leadership development.

Frequent thoughts about the value of experiential learning for ECRs and providing an enabling environment that included a critical mass of other research leaders from whom to learn were shared during the interviews and focus groups. There was a consensus that broader structural support was needed at the university level to create equitable and gender-sensitive models of research leadership training and research capacity building. Talent management was thought to be an important means to develop research leaders. Researchers required assistance in creating networks and gaining exposure with established researchers. Researchers – especially women – should be enabled to take up leadership opportunities. Researchers should also be given responsibilities, including teams to lead and other tasks targeted on developing their leadership potential such as brokering international opportunities. More small grants for early career researchers would provide further developmental opportunities.

Participants were asked to describe current practices and offer recommendations to increase the number of women research leaders in Africa. In terms of current practices, approaches to support women were limited to personal one-to-one supportive interventions. More strategic approaches or policies may exist at institutional level but were not referenced by participants. Regarding development approaches, participants offered wide-ranging recommendations for women researchers. The following were key views: (a) implementation of national policies to address marginalisation; (b) specific efforts to identify women with leadership potential and develop them; (c) promoting equal opportunity and recruitment through a quota system; (d) mainstreaming gender in all research activities and make including women mandatory in research funding; (e) and creating enabling environments and programmes.

Two survey respondents shared:

*“Institutional structures must provide equal and equitable support for researchers otherwise there will always be a gender imbalance.”*

*“Relevant employment equity policies – the institution, as a whole must subscribe to the principle of gender equity in the employment of all staff of whom potential research leaders are only a small subset.”*

The challenges identified by EMCRs commonly focused on gender pre-determined roles in the family and home, rather than issues in the research environment. Women were expected to ‘first sort out’ their home and social responsibilities and obligations: aspiring men research leaders did not have these constraints. Mindsets of women as well as men needed to change, so that women realise their leadership potential. It was contended that culture change must start at primary school to enable girls and young women to feel able to take up challenges. Suggestions for specific actions included postgraduate scholarships for women and dedicated mentoring programmes and other female-only schemes; special provision, such as when travel is required, provision for babies and nannies/larger rooms; gender-sensitive grant schemes.

## Conclusions

The article offers insights into the common career paths of EMCRs to research leadership, potential challenges facing ECRs and MCRs, general characteristics and competencies required to develop as a research leader and development approaches for future EMCRs. In line with Browning *et al*. (2017), the result of this study points to the urgent need for HEIs to capitalise and empower their ECRs as early as possible though networking opportunities, participation in conferences, establishing meaningful collaborations, mentorship, access to funding and supervision roles - providing a supportive and enabling research setting. The advancement of ECRs ultimately depend on opportunities for professional development, leadership training and access to resources. HEIs need to be deliberate and strategic on the efforts in developing the next generation of research leader. This requires planning, funding and commitment to ensure that ECRs are being given opportunities and support for scaling up to the responsibilities that DELTAS research leaders identified, and it needs to start early on. It is not just about training programs, it’s also about on the job experiences provided by the research leader to the next generation, recognition and support of key transition points as responsibilities scale up, - and so it comes full circle to current research leaders to concern themselves with the development of the next generation in an inclusive rather than exclusive way. And so, research leadership is truly about the research and the researcher. Especially in a context where researchers have concern for the common good, this is good starting point of developing a community as well as individuals.

Attracting, retaining, developing, and promoting the research leaders of the future is a priority that universities wishing to remain competitive cannot afford to ignore. Addressing this issue is vital to universities and will not only enable the development of human capital in the higher education sector, but it will also extend to excellence in research and teaching, and contribute to innovation and economic growth. With a better understanding of how the career paths of these research leaders developed, and how professional development programmes can support the next generation of researchers and research leaders, the fittest will not just be surviving, but thriving.

## Data availability

### Underlying data

Figshare: Career Transition to Research Leadership in Africa Qualitative Survey https://doi.org/10.6084/m9.figshare.14191682.v2 ( Viney & De-Graft Aikins, 2021a).

Figshare: Career Transition to Research Leadership in Africa Transcript - Focus Group 1 FINAL.pdf, https://doi.org/10.6084/m9.figshare.14191679.v1 ( Viney & De-Graft Aikins, 2021b).

Figshare: Career Transition to Research Leadership in Africa Transcript - Focus Group 2 FINAL.pdf, https://doi.org/10.6084/m9.figshare.14191697.v1 ( Viney & De-Graft Aikins, 2021c).

Figshare: Career Transition to Research Leadership in Africa Transcript - Interview 1.pdf, https://doi.org/10.6084/m9.figshare.14191688.v1 ( Viney & De-Graft Aikins, 2021d).

Figshare: Career Transition to Research Leadership in Africa Transcript - Interview 2.pdf, https://doi.org/10.6084/m9.figshare.14191685.v1 ( Viney & De-Graft Aikins, 2021e).

Figshare: Career Transition to Research Leadership in Africa Transcript - Interview 3.pdf, https://doi.org/10.6084/m9.figshare.14191691.v1 ( Viney & De-Graft Aikins, 2021f).

Figshare: Career Transition to Research Leadership in Africa Transcript - Interview 4.pdf, https://doi.org/10.6084/m9.figshare.14191709.v1 ( Viney & De-Graft Aikins, 2021g).

Figshare: Career Transition to Research Leadership in Africa Transcript - Interview 5.pdf, https://doi.org/10.6084/m9.figshare.14191724.v1 ( Viney & De-Graft Aikins, 2021h).

Figshare: Career Transition to Research Leadership in Africa Transcript - Interview 6.pdf, https://doi.org/10.6084/m9.figshare.14191694.v1 ( Viney & De-Graft Aikins, 2021i).

Figshare: Career Transition to Research Leadership in Africa Transcript - Interview 7.pdf, https://doi.org/10.6084/m9.figshare.14191700.v1 ( Viney & De-Graft Aikins, 2021j).

Figshare: Career Transition to Research Leadership in Africa Transcript - Interview 8.pdf, https://doi.org/10.6084/m9.figshare.14191706.v1 ( Viney & De-Graft Aikins, 2021k).

Figshare: Career Transition to Research Leadership in Africa Transcript - Interview 9.pdf, https://doi.org/10.6084/m9.figshare.14191712.v1 ( Viney & De-Graft Aikins, 2021l).

Figshare: Career Transition to Research Leadership in Africa Transcript - Interview 10.pdf, https://doi.org/10.6084/m9.figshare.14191703.v1 ( Viney & De-Graft Aikins, 2021m).

Figshare: Career Transition to Research Leadership in Africa Transcript - Interview 11.pdf, https://doi.org/10.6084/m9.figshare.14191721.v1 ( Viney & De-Graft Aikins, 2021n).

Figshare: Career Transition to Research Leadership in Africa Transcript - Interview 12.pdf, https://doi.org/10.6084/m9.figshare.14191715.v1 ( Viney & De-Graft Aikins, 2021o).

Figshare: Career Transition to Research Leadership in Africa Transcript - Interview 13.pdf, https://doi.org/10.6084/m9.figshare.14191718.v1 ( Viney & De-Graft Aikins, 2021p).

Figshare: Career Transition to Research Leadership in Africa Transcript - Interview 14.pdf, https://doi.org/10.6084/m9.figshare.14191727.v1 ( Viney & De-Graft Aikins, 2021q).

Data are available under the terms of the Creative Commons Attribution 4.0 International license (CC-BY 4.0).

## References

[ref-47] ACOLA: Career Support for Researchers: Understanding Needs and Developing a Best Practice Approach.2012;1–60. Reference Source

[ref-40] Baldwin R DeZure D Shaw A : Mapping the Terrain of Mid-Career Faculty at a Research University: Implications for Faculty and Academic Leaders. *Change.* 2008;40(5):46–55. 10.3200/CHNG.40.5.46-55

[ref-1] Bosanquet A Mailey A Matthews KE : Redefining ‘early career’ in academia: A collective narrative approach. *Higher Education Research & Development.* 2017;36(5):890–902. 10.1080/07294360.2016.1263934

[ref-3] Browning L Thompson K Dawson D : From early career researcher to research leader: Survival of the fittest? *Journal of Higher Education Policy and Management.* 2017;39(4):361–377. 10.1080/1360080X.2017.1330814

[ref-4] Clay M : Sink or swim: Drowning the next generation of research leaders? *Australian Institute of Policy and Science.* 2012;83(4):26–31. Reference Source

[ref-49] Dever M Morrison Z Dalton B : When Research Works for Women Project Report.2006;1–48. Reference Source

[ref-5] European Commission: Towards a European Framework for Research Careers: Mobility patterns and career paths of researchers (MORE _3_) study.2018. Reference Source

[ref-43] Evans L : What is Effective Research Leadership? A Research Informed Perspective. *Higher Education Research & Development.* 2014;33:1, 46–58. 10.1080/07294360.2013.864617

[ref-44] Fraser K Greenfield R Pancini G : Conceptualising Institutional Support for Early, Mid, and Later Career Teachers. *International Journal for Academic Development.* 2017;22(2):157–169. 10.1080/1360144X.2016.1218882

[ref-6] Frick L Albertyn R Brodin E : The role of doctoral education in early career academic development.In M. Fourie- Malherbe, C. Aitchison, E. Blitzer and R. Albertyn (eds.) *Postgraduate Supervision: Future foci for the knowledge society.*Stellenbosch, Sun Press:2017;203–219. 10.18820/9781928357223/12

[ref-7] Harle J : Foundations for the future: Supporting the early careers of African researchers.2011;4–35. Reference Source

[ref-8] Hottenrott H Lawson C : Flying the nest: How the home department shapes researchers' career paths. *Studies in Higher Education.* 2017;42(6):1091–1109. 10.1080/03075079.2015.1076782

[ref-9] Hurley J Taylor EJ : Australian early career planning researchers and the barriers to research–practice exchange. *Australian Planner.* 2016;53(1):5–14. 10.1080/07293682.2015.1135813

[ref-41] Merritt C Jack J Mangezi W : Positioning for Success: Building Capacity in Academic Competencies for Early-Career Researchers in Sub-Saharan Africa. *Glob Ment Health (Camb).* 2019;6:e16. 10.1017/gmh.2019.14 31391948 PMC6669964

[ref-52] Moosa R : Early Career Academic Development And Talent Management In the South African Higher Education Context. Higher Education Research and Development Society of Australasia Inc.,2020;73–84. Reference Source

[ref-42] Owusu F Kalipeni E Kiiru JMM : Capacity Building for Research Leadership: The Need, Support and Strategies for Growing African Research Leaders. *Partnership for African Social & Governance Research.* 2014;1–64. Reference Source

[ref-45] Richards GC Bradley SH Dagens AB : Challenges facing Early-Career And Mid-Career Researchers: Potential Solutions to Safeguard the Future of Evidence-Based Medicine. *EBM Opinion and Debate: General Medicine.* 2021;26(1):8–11. 10.1136/bmjebm-2019-111273 31653688

[ref-14] Schriever V Grainger P : Mentoring an early career researcher: Insider perspectives from the mentee and mentor. *Reflective Practice.* 2019;20(6):720–731. 10.1080/14623943.2019.1674272

[ref-15] Shinkafi TS : Challenges experienced by early career researchers in Africa. *Future Sci OA.* 2020;6(5):FSO469. 10.2144/fsoa-2020-0012 32518684 PMC7273411

[ref-16] Sullivan SE Ariss AA : Making sense of different perspectives on career transitions: A review and agenda for future research. *Human Resource Management Review.* 2021;31(1):100727. 10.1016/j.hrmr.2019.100727

[ref-50] UNESCO: UNESCO and Gender Equality in Sub-Saharan Africa: Innovative Programmes, Visible Results. UNESCO,2017;5–94. Reference Source

[ref-17] Viney C De-Graft Aikins A : Career Transition to Research Leadership in Africa Qualitative Survey. *figshare.* Dataset.2021a. 10.6084/m9.figshare.14191682.v2 PMC8323068 34381872

[ref-18] Viney C De-Graft Aikins A : Career Transition to Research Leadership in Africa Transcript - Focus Group 1 FINAL.pdf. *figshare.* Dataset.2021b. 10.6084/m9.figshare.14191679.v1 PMC8323068 34381872

[ref-19] Viney C De-Graft Aikins A : Career Transition to Research Leadership in Africa Transcript - Focus Group 2 FINAL.pdf. *figshare.* Dataset.2021c. 10.6084/m9.figshare.14191697.v1 PMC8323068 34381872

[ref-20] Viney C De-Graft Aikins A : Career Transition to Research Leadership in Africa Transcript - Interview 1.pdf. *figshare.* Dataset.2021d. 10.6084/m9.figshare.14191688.v1 PMC8323068 34381872

[ref-21] Viney C De-Graft Aikins A : Career Transition to Research Leadership in Africa Transcript - Interview 2.pdf. *figshare.* Dataset.2021e. 10.6084/m9.figshare.14191685.v1 PMC8323068 34381872

[ref-22] Viney C De-Graft Aikins A : Career Transition to Research Leadership in Africa Transcript - Interview 3.pdf. *figshare.* Dataset.2021f. 10.6084/m9.figshare.14191691.v1 PMC8323068 34381872

[ref-23] Viney C De-Graft Aikins A : Career Transition to Research Leadership in Africa Transcript - Interview 4.pdf. *figshare.* Dataset.2021g. 10.6084/m9.figshare.14191709.v1 PMC8323068 34381872

[ref-24] Viney C De-Graft Aikins A : Career Transition to Research Leadership in Africa Transcript - Interview 5.pdf. *figshare.* Dataset.2021h. 10.6084/m9.figshare.14191724.v1 PMC8323068 34381872

[ref-25] Viney C De-Graft Aikins A : Career Transition to Research Leadership in Africa Transcript - Interview 6.pdf. *figshare.* Dataset.2021i. 10.6084/m9.figshare.14191694.v1

[ref-26] Viney C De-Graft Aikins A : Career Transition to Research Leadership in Africa Transcript - Interview 7.pdf. *figshare.* Dataset.2021j. 10.6084/m9.figshare.14191700.v1

[ref-27] Viney C De-Graft Aikins A : Career Transition to Research Leadership in Africa Transcript - Interview 8.pdf. *figshare.* Dataset.2021k. 10.6084/m9.figshare.14191706.v1

[ref-28] Viney C De-Graft Aikins A : Career Transition to Research Leadership in Africa Transcript - Interview 9.pdf. *figshare.* Dataset.2021l. 10.6084/m9.figshare.14191712.v1

[ref-29] Viney C De-Graft Aikins A : Career Transition to Research Leadership in Africa Transcript - Interview 10.pdf. *figshare.* Dataset.2021m. 10.6084/m9.figshare.14191703.v1

[ref-30] Viney C De-Graft Aikins A : Career Transition to Research Leadership in Africa Transcript - Interview 11.pdf. *figshare.* Dataset.2021n. 10.6084/m9.figshare.14191721.v1

[ref-31] Viney C De-Graft Aikins A : Career Transition to Research Leadership in Africa Transcript - Interview 12.pdf. *figshare.* Dataset.2021o. 10.6084/m9.figshare.14191715.v1

[ref-32] Viney C De-Graft Aikins A : Career Transition to Research Leadership in Africa Transcript - Interview 13.pdf. *figshare.* Dataset.2021p. 10.6084/m9.figshare.14191718.v1

[ref-33] Viney C De-Graft Aikins A : Career Transition to Research Leadership in Africa Transcript - Interview 14.pdf. *figshare.* Dataset.2021q. 10.6084/m9.figshare.14191727.v1

[ref-34] Vitae : What do researchers do - Exploring the destinations and career paths of doctoral graduates and how they contribute to society, culture and economy. 2020. Reference Source

[ref-35] Vitae : The Vitae Researcher Development Framework (RDF). 2012. Reference Source

[ref-36] Williams B Christensen E Occhino J : Tinkering through transition on doctoring as an early-career academic in physical education and sport pedagogy. *Sport Educ Soc.* 2017;22(1):73–86. 10.1080/13573322.2016.1260540

[ref-37] Wong N : The changing landscapes of research funding: Challenges for mid -career researcher. 2019. 10.1186/s13059-019-1798-9 PMC6712669 31462298

