# Supplementary material for: Early- and mid-career transitions to research leadership in Africa
Source: Wellcome Open Res. 2021 Jul 23;6:74. Originally published 2021 Apr 7. [Version 2] doi: 10.12688/wellcomeopenres.16540.2 (PMC8323068; doi:10.12688/wellcomeopenres.16540.2)
Supplement: Peer review history

# Peer review: Reviewer response for version 2

**DOI:** 10.21956/wellcomeopenres.18856.r45173

**Published:** 2021-08-12

**Authors:** Jeanette Maritz

**Affiliations:** 1 Department of Health Studies, University of South Africa (Unisa), Pretoria, South Africa

This article focuses on early-and mid-career transitions in the Global South and contributes to the African continent's body of knowledge and voices on research leadership, career transitions and challenges regarding research training. The African context is unique due to its diversity of cultural beliefs, practices, infrastructure, systems and resources.

 The authors open with a claim that concepts of early career researchers (ECRs) and mid-career researchers (MCRs) lack a universal understanding and are often defined differently in the Global North versus the Global South. The Global North conceptualise the transition of ECRs to research leaders by acquiring research capital, leading and influencing large research teams and having sizable grants. In contrast, Universities on the African continent define ECP categories to post descriptions such as progression from lecturers to professors and Australian research and universities according to years after completing a doctoral degree. A difference is also marked between men and women, their career trajectories and the time to focus on their career paths. Each transition from ECP to MCR to research leader poses specific and often unique challenges.

 A mixed methods approach was appropriately used to triangulate the data. Qualitative data were collected using individual interviews (n=24) and focus groups (n=27) to identify key transition points of early career researchers (ECRs) and mid-career researchers (MCRs) in selected African countries. The qualitative data were complemented with quantitative survey questionnaires (n=250). The article offers insights into the typical career paths of EMCRs to research leadership, potential challenges facing ECRs and MCRs, general characteristics and competencies required to develop as a research leader and development approaches for future EMCRs.

 A critical finding is the importance of career ownership, self-management and planning from the outset of one's career, being proactive and making intentional decisions towards becoming a research leader. It requires acquiring new competencies as one progresses through the different phases and taking on new and often more significant responsibilities, including being responsible for a team. Targeted training and support in the form of mentors, funding, networking opportunities, to name a few, are needed within an enabling research environment for each career junction. In addition to the interventions suggested by the authors, Vice (2010, 34) ^1^ indicates that ''race is the unacknowledged elephant in the room that affects pretty much everything in and outside academia''. Identity politics and relational ontologies are equally emerging as aspects to be noted in the process of becoming research leaders (Maritz & Prinsloo, 2021) ^2^, and research developers could take note of these aspects in their offerings of support. This will allow researchers to prosper and universities retain their critical skills to lead the next generation of leaders.

Is the work clearly and accurately presented and does it cite the current literature?

Yes

If applicable, is the statistical analysis and its interpretation appropriate?

Yes

Are all the source data underlying the results available to ensure full reproducibility?

Yes

Is the study design appropriate and is the work technically sound?

Yes

Are the conclusions drawn adequately supported by the results?

Yes

Are sufficient details of methods and analysis provided to allow replication by others?

Yes

Reviewer Expertise:

Early to mid-career research development and support

I confirm that I have read this submission and believe that I have an appropriate level of expertise to confirm that it is of an acceptable scientific standard.

## References

[1] : “How Do I Live in This Strange Place?”. *Journal of Social Philosophy*.2010;41(3) : 10.1111/j.1467-9833.2010.01496.x323-34210.1111/j.1467-9833.2010.01496.x

[2] : Narrating the (dis)comfort and multiplicities of becoming faculty. *Gender and Education*.2021;33(6) : 10.1080/09540253.2020.1860198692-70610.1080/09540253.2020.1860198

# Peer review: Reviewer response for version 2

**DOI:** 10.21956/wellcomeopenres.18856.r45176

**Published:** 2021-07-29

**Authors:** Hamid R Jamali

**Affiliations:** 1 School of Information Studies, Charles Sturt University, Wagga Wagga, NSW, 2678, Australia

The article, using a mixed-method approach, presents some interesting findings about early and mid-career researchers in part of Africa and their transitions to research leadership. Given there isn't much research on this part of the world, the paper is a good addition to the literature.

 While the paper is fine I think it can benefit from the clarification of a few things as I suggest below. 

 While interviews, focus groups and a (quantitative) survey have been used in the study, and triangulation has been done, there are really few findings from the survey incorporated in the paper, except from two quotations perhaps from participants' responses to open-ended questions. So it should be clarified earlier in the paper that the focus of the paper seems to be the qualitative data and findings. 

 The method says the survey was distributed to 250 respondents and given the abstract says (n=250) it gives the impression that the response rate was 100% which is rarely the case in surveys. This needs to be clarified. if the survey was distributed to more people and only 250 usable responses were collected, the statement in methods needs to be corrected. 

 Please add the word 'survey' to the title of Figure 1 for clarity (i.e. African countries where survey respondents were working).

 The results section could benefit from more categorisation. The same way that the results in the 'General characteristics...' section have been categorised and visualised, some sort of list or categories for sections on 'Challenges...' or 'Common career paths...' would be useful. I think that would make the paper easier to read for readers. 

 It would be useful if the authors mention the source of findings in each case. In some cases, they mentioned the findings was an outcome of the focus group or interview and in some cases that is not clear. If authors think this would be useful, they might consider adding that information.

 Finally, given the richness of the data that have been collected for this project (interview, focus group, etc.), I expected the paper to be more extensive with more findings and details. It might be the case the more publications hopefully will come out of the project.

Is the work clearly and accurately presented and does it cite the current literature?

Yes

If applicable, is the statistical analysis and its interpretation appropriate?

Not applicable

Are all the source data underlying the results available to ensure full reproducibility?

Yes

Is the study design appropriate and is the work technically sound?

Yes

Are the conclusions drawn adequately supported by the results?

Yes

Are sufficient details of methods and analysis provided to allow replication by others?

Partly

Reviewer Expertise:

Research evaluation, scholarly communication

I confirm that I have read this submission and believe that I have an appropriate level of expertise to confirm that it is of an acceptable scientific standard.

# Peer review: Reviewer response for version 1

**DOI:** 10.21956/wellcomeopenres.18226.r44348

**Published:** 2021-07-05

**Authors:** Julie Davies

**Affiliations:** 1 Manchester Metropolitan University Business School, Manchester Metropolitan University, Manchester, UK

This article provides very useful insights from a survey into research leadership in Africa, specifically focusing on transitions for doctoral students, early and mid career researchers based on the vitae researcher development model. It highlights common career paths and challenges at individual levels as well as in university systems. The article recommends further research on career paths of research leaders to support future generations. Specific support is suggested, e.g. mentoring, experiential learning, networking, grants, 1-1 support for women. There are clear equity, diversity and inclusion policy issues to mitigate gender inequalities in the African researcher community.

 International collaboration is a key recommendation and it will be interesting to see examples of successful models to support talent management and capacity building. 

 Quality of written English is generally fine. Please note the following:

**Early- and mid-career transition to research leadership in Africa – change to transitions**

 the early-and mid-career transition to research leadership in Africa. Much of the available African literature on research leadership indicate

 while the African literature classify ECRs

 provided insight

 Given that no universally accepted definition for ECRs exist,

 Research on MCRs indicate 

 doctorate enrolments as a proportion of all HE enrolments has

 A senior research leader who was intentional about career ownership and planning shared: - replaced shared with commented

 On the later stages – change to During the

 can be categorised as follow: should be follows

 This is not an emerging theme and have surfaced in the existing international literature for some time – change to it has surfaced in existing

 The advancement of ECRs ultimately depend on – change to depends on

 deliberate and strategic on the efforts in developing the next generation of research leader. Change to: strategic about the … research leaders

 this is good starting point of developing a community as well as individuals. Changed to a good starting point for

Is the work clearly and accurately presented and does it cite the current literature?

Partly

If applicable, is the statistical analysis and its interpretation appropriate?

Not applicable

Are all the source data underlying the results available to ensure full reproducibility?

Yes

Is the study design appropriate and is the work technically sound?

Yes

Are the conclusions drawn adequately supported by the results?

Yes

Are sufficient details of methods and analysis provided to allow replication by others?

Yes

Reviewer Expertise:

Research impact policy evaluation, EDI, higher education management and internationalisation.

I confirm that I have read this submission and believe that I have an appropriate level of expertise to confirm that it is of an acceptable scientific standard.
